# Retraction: ATP Mediates NADPH Oxidase/ROS Generation and COX-2/PGE_2_ Expression in A549 Cells: Role of P2 Receptor-Dependent STAT3 Activation

**DOI:** 10.1371/journal.pone.0268877

**Published:** 2022-05-19

**Authors:** The PLOS ONE Editors

Following the publication of this article [1], concerns were raised regarding results presented in multiple figures. Specifically,

- In Fig 2E, the following panels appear to partially overlap despite representing different experimental conditions:
  ○ The 0 min and 5 min panels.
  ○ The 30 min and 60 min panels.
- In Figs 2F, 3B and 4D, the Basal and ATPγS control panels appear similar.
- The following western blot data appear similar despite representing different conditions:
  ○ Lanes 3–5 of the p47^*phox*^ panel in Fig 2G, lanes 1–3 of the PKCα membrane panel in Fig 4F flipped horizontally, and lanes 1–3 of the PKCί membrane panel in Fig 4F.
  ○ Lane 2 of the p47^*phox*^ panel in Fig 2G and lane 4 of the PKCα membrane panel in Fig 4F, flipped horizontally.
  ○ Lane 6 of the p47^*phox*^ panel in Fig 2G and lane 4 of the PKCί membrane panel in Fig 4F.
  ○ Lane 1 of the GAPDH panel and lane 4 of the Gαs panel in Fig 4F.
  ○ The COX-2 panel for PPADS in Fig 4A and the COX-2 panel for CBE in Fig 5A.
  ○ In Fig 5F, the Jak2 and STAT3 panels, and the β-actin for Jak2 and β-actin for STAT3 panels.
  ○ In Fig 6A (right side), lanes 1–2 of the T-STAT3 (CE) panel and lanes 6–7 of the T-STAT3 (NE) panel.

The corresponding author stated that the 5 min and 60 min panels in Fig 2E were incorrect. They provided an updated figure with these panels replaced and raw image data underlying this experiment.

The corresponding author stated that multiple western blot experiments were performed and imaged at the same time. They acknowledged that errors occurred in the preparation of the figures and annotation of raw images, and apologized for these errors. The corresponding author accepted that the issues with the published figures cannot be fully resolved, and requested that the article be retracted.

In light of the concerns affecting multiple figure panels and the errors in the raw underlying data that question the reliability of these data, the *PLOS ONE* Editors retract this article.

ITL, CCL, WLW, LDH and CMY agreed with the retraction and apologize for the issues with the published article. SEC either did not respond directly or could not be reached.
